# Anticancer and Antioxidant Activity of Water-Soluble Polysaccharides from *Ganoderma* aff. *australe* against Human Osteosarcoma Cells

**DOI:** 10.3390/ijms232314807

**Published:** 2022-11-26

**Authors:** Tatiana Muñoz-Castiblanco, Lucía Santa Maria de la Parra, Rocio Peña-Cañón, Juan Camilo Mejía-Giraldo, Ignacio E. León, Miguel Ángel Puertas-Mejía

**Affiliations:** 1 Grupo de Investigación en Compuestos Funcionales, Facultad de Ciencias Exactas y Naturales, Universidad de Antioquia (UdeA), Calle 70 No. 52-21, Medellín 050010, Colombia; tatiana.munoz2@udea.edu.co (T.M.-C.); juan.mejia8@udea.edu.co (J.C.M.-G.); 2 Centro de Química Inorgánica, (CEQUINOR UNLP, CCT-CONICET La Plata, Asociado a CIC), Departamento de Química, Facultad de Ciencias Exactas, Universidad Nacional de La Plata, Blvd. 120 No. 1465, La Plata 1900, Argentina; luciasmdlp@gmail.com (L.S.M.d.l.P.); ileon@biol.unlp.edu.ar (I.E.L.); 3 Grupo de Investigación Biología para la Conservación, Escuela de Ciencias Biológicas, Facultad de Ciencias Básicas, Universidad Pedagógica y Tecnológica de Colombia, Avenida Central del Norte 39-115, Tunja 150003, Colombia; erociopc030987@gmail.com; 4 Facultad de Ciencias Farmacéuticas y Alimentarias, Universidad de Antioquia (UdeA), Calle 70 No. 52-21, Medellín 050010, Colombia; 5 Cátedra de Fisiopatología, Departamento de Ciencias Biológicas, Facultad de Ciencias Exactas, Universidad Nacional de La Plata, 47 y 115, La Plata 1900, Argentina

**Keywords:** anticancer activity, antioxidant activity, *G.* aff. *australe*, MG-63 cells, mushrooms, osteosarcoma, polysaccharides

## Abstract

Wild mushrooms have gained great importance for being a source of biologically active compounds. In this work, we evaluate the anticancer and antioxidant activity of a water-soluble crude polysaccharide extract isolated from the fruiting bodies of the *Ganoderma* aff. *australe* (GACP). This mushroom was collected in San Mateo (Boyacá, Colombia) and identified based on macroscopic and microscopic characterization. GACP was characterized by UV–Vis spectroscopy, Fourier-transform infrared spectroscopy, high-performance liquid chromatography–diode array detector, and nuclear magnetic resonance. The antiradical and antioxidant activity were evaluated by different methods and its anticancer activity was verified in the osteosarcoma MG-63 human cell line. Chemical and spectroscopic analysis indicated that GACP consisted of β-D-Glcp-(1→, →3)-β-D-Glcp-(1→ and α-D-Glcp-(1→ residues. The results of the biological activity showed that GACP exhibited high antioxidant activity in the different methods and models studied. Moreover, the results showed that GACP impaired cell viability (3-(4,5-dimethylthiazol-2-yl)-2,5-diphenyltetrazolium bromide (MTT) assay) and cell proliferation (clonogenic assay) in a dose–response manner on MG-63 cells. The findings of this work promote the use of mushroom-derived compounds as anticancer and antioxidant agents for potential use in the pharmaceutical and food industries.

## 1. Introduction

The fungi kingdom is one of the most diverse resources, which have important functions in ecosystems. In addition, they are a source of bioactive metabolites with potential use in the field of biomedical sciences and the food industry [1,2]. Especially in Colombia, wild mushrooms have a lot of traditional applications in rural communities from the northeastern Andes, such as food, medicine, and also as a cultural factor [3]. Despite the relevance of this ecological resource, there are only a few studies based on the compounds extracted from these mushrooms, as potential antioxidant and anticancer agents [4,5].

The *Ganoderma* genus has been widely studied in the world for its important medicinal properties derived from its bioactive compounds. Among these, polysaccharides are one of the most abundant components with important biological activities, including antioxidant, antibacterial, immunomodulatory, and anticancer activity, among others [6,7]. The antioxidant activity of polysaccharides is of special interest since they reduce oxidative stress, induced by an excess of reactive oxygen species (ROS). Thus, polysaccharides could mitigate the toxicities associated with overexpression of ROS and provide a potential therapeutic approach in the prevention of various human diseases [8,9]. Moreover, the interest in studying the biological activity of mushroom polysaccharides has increased, since it has been shown that they have anticancer activities or can improve the efficacy of conventional cancer treatments [10,11]. Three possible mechanisms by which polysaccharides have antitumor activity have been reported: (a) indirect activity through improving immunity function; (b) direct activity through inhibiting the growth of cancer cells; and (c) acting as a chemotherapy adjuvant [12].

Osteosarcoma is the most common primary solid bone tumor and it is one of the most frequent causes of cancer death in childhood. It mainly affects long bones which grow rapidly in childhood and adolescence. Although surgical resection in combination with radiotherapy or systemic chemotherapy improves the survival rate of patients with osteosarcoma, it lacks total effectiveness and could generate harmful effects [13,14]. Additionally, many patients develop resistance to chemotherapy [13], so it is necessary to search for other alternatives that allow the counteracting of these disadvantages. Some authors have reported that *Ganoderma lucidum* [15] and *Trametes robiniophila* Murrill [16] polysaccharides exhibited in vivo cytotoxic effects on human osteosarcoma cells. The results showed that these polysaccharides modulated the expression of Bcl-2, Bax, and the release of cytochrome c from the mitochondria. This work reports, for the first time, the characterization and evaluation of the anticancer and antioxidant activity of the water-soluble crude polysaccharide extract isolated from the fruiting bodies of *Ganoderma* aff. *australe*. We also demonstrate that *G.* aff. *australe* has the potential to scavenge some radicals and reduce cell proliferation in a concentration-dependent manner.

## 2. Results and Discussion

### 2.1. Taxonomic Identification of Specimens

#### *Ganoderma* aff. *australe* (Fr.) Pat. 1889

**Figure 1:**
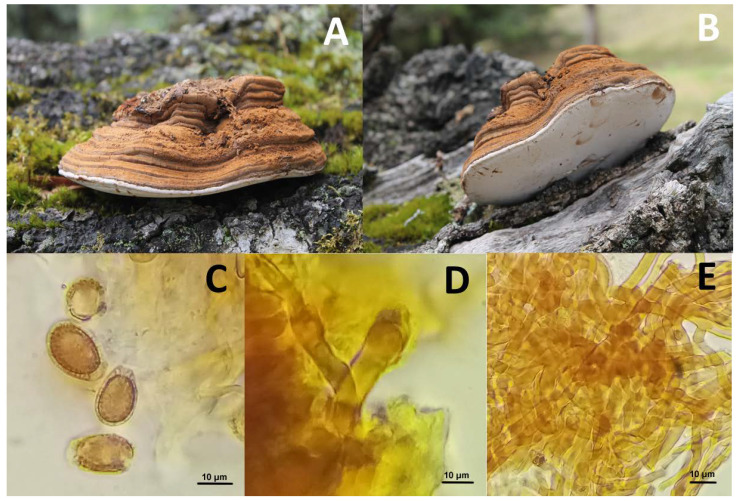
*Ganoderma* aff. *australe*. Appearance of Basidiome (**A**), Hymenophore (**B**), Basidiospores (**C**), Basidia (**D**), Hyphae (**E**). Scale bar = 10 µm (100×), binocular microscope. Font: (**A**,**B**) Rocio Peña-Cañón; (**C**–**E**) Yeina Niño.

*Basidiome*: sessile, non-laccate, woody. *Pileus*: 17 cm in diameter, flattened, planoconvex to sub-orbicular; upper surface opaque, concentrically furrowed, with an irregular bark covering the cap, dark orange brownish in the center becoming darker towards the margin, with concentric lines of a lighter tone; margin soft, slightly lobed and concolorous with the rest pileus. *Context:* 0.2 to 2 cm thick increasing towards the base of attachment to the substrate, dry, hard to slightly leathery, dark reddish brown, musty odor, indeterminate taste (Figure 1A). *Hymenophore:* tubular, composed of tubes, 1.8 cm long, dark brown, semicircular to circular pores towards the margin, approximately two per mm, white oxidizing to dark reddish brown when touched or handled. Spore prints dark reddish brown (Figure 1B).

Habitat: Scattered to gregarious, on a decaying wood log in grassland at the edge of an oak forest (*Quercus humboldtii* Bonpl.).

*Basidiospores:* 12–14 × 5–6 µm (*n* = 10), ellipsoid, ornamented, reddish-brown in 10% KOH, guttulate, without obvious germinal pore, inamyloid in Melzer, slightly thick double-walled, slightly echinulate inner wall and the external smooth, covered by a hyaline exosporium (Figure 1C); *Basidia*: 15–20 × 3–4 µm, cylindrical, 4-sterigmate, thin-walled, inamyloid in Melzer (Figure 1D); *Context:* trimitic, interwoven, generative hyphae, 4 µm diameter, thin-walled, brown in 10% KOH, inamyloid in Melzer; thick-walled, orange-brown, skeletal hyphae and union hyphae, branching, thick-walled, brown, inamyloid in Melzer (Figure 1E).

Specimen examined—COLOMBIA, Boyacá. Municipality of San Mateo, La Peñuela village, Alto del roble local; 5°36’17.6” N, 73°56’0.85” W; 2698 m above sea. Date: 12 October 2019. Collectors: Peña-Cañón, R.|Niño-Fernández, Y.|Rosero, L.—1255 (HUA-228455). The species has been reported for the departments of Amazonas, Caquetá, and Chocó at an elevation between 50–200 m above sea level [17] and Cundinamarca between 2568 and 2584 m above sea level [18] and the present record for the department of Boyacá.

Comments—*G. australe* (Fr.) Pat. 1889 (*Polyporaceae*), belongs to the *G. applanatum-australe* complex, can be microscopically distinguished from *G. applanatum* by having larger basidiospores. In Cundinamarca, Pinzón-Osorio and Pinzón-Osorio (2016) cite spores with sizes (6.2–13.3 × 4–7.5 µm, Q = 1.2–1.6) similar to specimens collected in China ((6.1) 7.6–10.8 (11.5) × (4.7) 5.3–7.9 (8.5) μm (Q = 1.5–2.6)) and identified using classical taxonomy and molecular phylogeny (ITS). This species is common in the tropics and phylogenetically distant from *G. applanatum* [19].

### 2.2. Characterization of GACP

#### 2.2.1. Extraction and Chemical Composition

The extraction yield of the GACP was 1.05% of the mushroom dry weight. This result is higher than that reported for *G. lucidum* (0.5%) and *G. applanatum* (0.2%) [20]. However, these yields could be related to the dialysis and precipitation process, since it has been reported that they allow the removal of 50–90% of free soluble carbohydrates and small compounds from the extract. These carbohydrates can be found as a product of degradation processes during extraction and purification [20,21]. The chemical composition of GACP is shown in Table 1.

Even though processes that allow the elimination of low molecular weight compounds such as precipitation with ethanol and dialysis were used to obtain GACP, 3.49 ± 0.06% of phenolic compounds were found in GACP. This may be related to the presence of interactions between high molecular weight polysaccharides and phenolic compounds, due to the ability of the latter to form hydrophobic interactions or hydrogen bonds with polysaccharides [22]. Moreover, the proteins present in GACP could be forming polysaccharide–protein complexes and interacting through covalent bonds [23]. The content of uronic acid and sulfate sugars in GACP was similar to those reported for *G. applanatum* polysaccharides [24]. 

**Figure 2:**
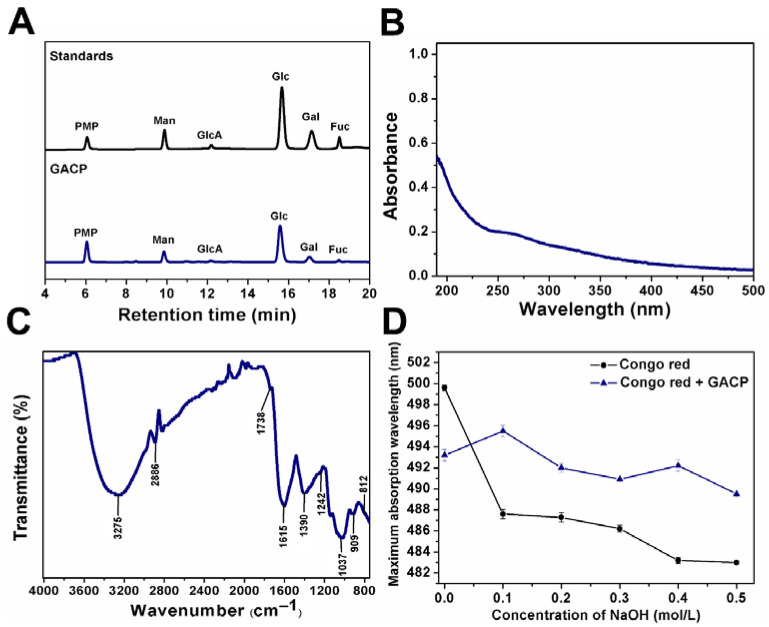
Characterization of GACP: HPLC chromatogram profiles of PMP derivates of monosaccharide standards (Man: mannose; GlcA: glucuronic acid; Glc: glucose; Ga: galactose; and Fuc: fucose) and hydrolysate of GACP (**A**), UV–Vis spectrum (**B**), FT-IR spectrum (**C**) and Congo red test (**D**).

Determining the composition of monosaccharides is important since it has been found that polysaccharides with glucose have better antioxidant activity than those that do not [25]. The HPLC chromatogram profile of monosaccharide composition is shown in Figure 2A. GACP is mainly composed of Glc, Man, Gal with low content of Fuc, and uronic acid, which demonstrated the high neutral sugar content (see Table 1). GACP is mainly composed of high molecular weight polysaccharides (>100 kDa: 55%, 50–100 kDa: 5%, and 10–50 kDa: 40%), so, according to literature reports, it could contain more side chains. In this way, there would be greater availability of the O-H and C-H groups that could contribute to antioxidant activity [26].

It has been reported that factors such as extraction conditions and method [6,27], age, and stage of development of the mushroom, among others [28], influence the type and content of bioactive compounds that can be found in the mushrooms. For this reason, the content of sugars, uronic acids, proteins, type, and proportion of monosaccharides of GACP varied with respect to that reported for other polysaccharides extracted from mushrooms of the *Ganoderma* genus [24,27,29,30,31,32].

#### 2.2.2. UV–Vis and FT-IR Spectrum Analysis of GACP

Figure 2B shows the UV–Vis spectrum of GACP. The absorption band between 260 and 280 nm, commonly related to the presence of phenols and proteins, is in agreement with the obtained chemical composition [33,34]. Thus, GACP could exhibit high antioxidant activity, since it has been reported that the antioxidant ability of polysaccharides is enhanced by the presence of proteins and phenols in their structure or as part of the crude extract [35].

On the other hand, the FT-IR spectrum of GACP showed characteristic absorption bands of polysaccharides (see Figure 2C). The broad absorption band at 3275 cm^−1^ was attributed to O-H stretching vibration related to intramolecular and intermolecular hydrogen bonds of the polysaccharide chains [36]. The absorption at 2886 cm^−1^ was assigned to C-H stretching vibration and absorption of 1738 cm^−1^ to carbonyl-group bending vibration, which suggested the presence of uronic acid in GACP [36,37]. The absorption band at 1615 cm^−1^ corresponded to C=O and C=C stretching vibrations and was attributed to the presence of protein and aromatic phenolic compounds. The band at 1390 cm^−1^ was attributed to OH groups of phenolic compounds [20]. The absorption bands at 1242 and 1037 cm^−1^ were related to the vibration of S=O in the sulfate group and the presence of pyranose rings, respectively [38,39]. These results are in agreement with the chemical composition results. In addition, the bands at 909 and 812 cm^−1^ were associated with the presence of β and α-configurations in GACP, respectively [40,41,42].

#### 2.2.3. Preliminary Results of GACP Conformation

The triple-helical conformation of polysaccharides is related to their biological activity [43]. In alkaline solutions, Congo red can form complexes with polysaccharides with a triple helical structure, resulting in a shift in maximum wavelength absorption (λ_max_) and a redshift. In addition, it has been shown that the hydrogen bonds that maintain the triple helical conformation are broken at high concentrations of NaOH, showing a reduction in the value of λ_max_ [44,45]. The interaction of Congo red with GACP is shown in Figure 2D. The highest λ_max_ was observed when the NaOH concentration was 0.1 mol/L and decreased when the NaOH concentration was 0.2 mol/L. This indicated that GACP has shown a triple helical structure, which was destroyed when the NaOH concentration increased. Likewise, it has been reported that *Stropharia rugosoannulata* and *Pleurotus eous* polysaccharides exhibit a triple helical conformation at low NaOH concentrations [46,47].

#### 2.2.4. NMR Analysis

**Figure 3:**
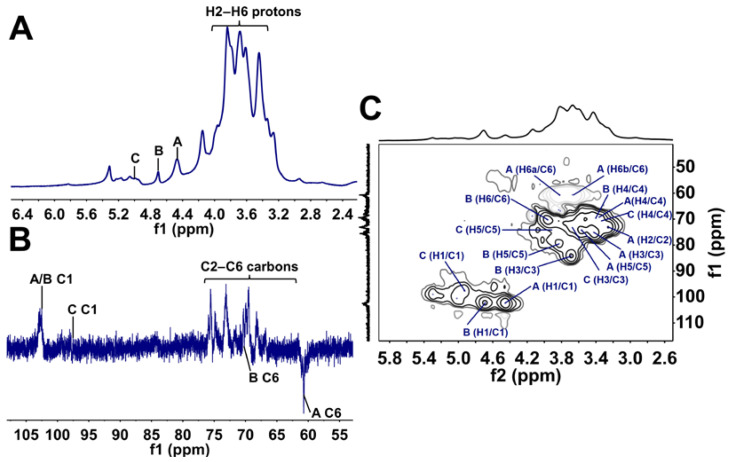
^1^H NMR (**A**), DEPT-135 (**B**) and ^1^H-^13^C HSQC (**C**) spectra of GACP. A, B and C refer to the residues found in the structure of GACP. A: β-D-Glcp-(1→; B: →3)-β-D-Glcp-(1→ and C: α-D-Glcp-(1→.

^1^H, DEPT-135, ^1^H-^13^C HSQC spectra are shown in Figure 3. Characteristic signals for each residue found in GACP were assigned based on the literature values [31,32,48,49]. In the ^1^H NMR spectrum of GACP (Figure 3A), three anomeric signals were observed between 4.40 and 5.00 ppm, indicating the presence of β (4.46 and 4.68 ppm) and α (4.93 ppm) configuration for glucopyranosyl residues. This results were according to the results of FTIR spectrum analysis. Additionally, in the DEPT-135 spectrum (Figure 3B), two signals in the anomeric region at 102.41 and 97.34 ppm were identified, which confirmed the presence of β and α configuration for glucopyranosyl residues. In the ^1^H-^13^C HSQC spectrum (Figure 3C), three cross-signals (A, B and C) were observed at 4.46/102.41, 4.68/102.41, and 4.93/97.34 ppm. They were assigned to H1/C1 of β-D-Glcp-(1→, →3)-β-D-Glcp-(1→ and α-D-Glcp-(1→, respectively, (see Table 2). Moreover, the DEPT-135 spectrum allowed confirmation of the C-6 linkages of residues in the HSQC spectrum.

### 2.3. Antioxidant Activity

Table 3 shows the results of antiradical activity of GACP and AA expressed as IC_50_ value (mg of compound/mL).

#### 2.3.1. ABTS and DPPH Radical Scavenging Ability

**Figure 4:**
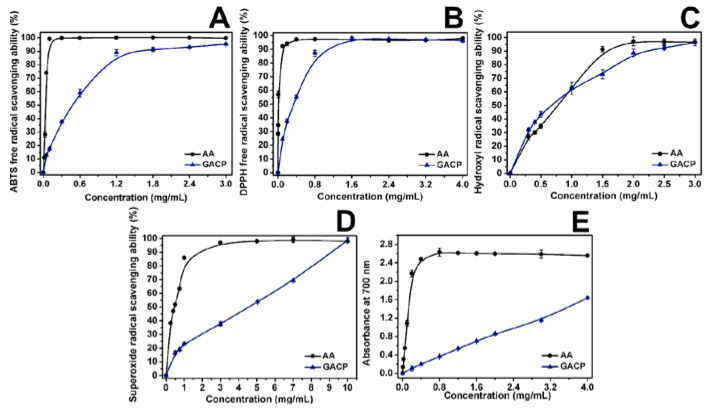
Antiradical activity of AA and GACP: ABTS radical assay (**A**); DPPH radical assay (**B**); hydroxyl radical assay (**C**); superoxide radical assay (**D**); reducing power assay (**E**). AA: ascorbic acid; ABTS: 2,2-azino-bis-(3-ethyl-benzthia-6-sulfonic acid); DPPH: 1,1-diphenyl-2-picrylhydrazyl.

The ABTS radical scavenging rate of GACP is shown in Figure 4A and increased in a concentration-dependent manner. AA exhibited high radical scavenging ability (99.92%) at 1.2 mg/mL, which was close to that of GACP (88.98%). However, compared with AA the radical scavenging ability of GACP was lower in concentrations between 0.1 and 0.6 mg/mL. An IC_50_ value for GACP of 0.48 mg/mL was found, which is better compared to that reported for *Pleurotus dmajor* (0.82 mg/mL) polysaccharides [50]. This demonstrated the great potential of GACP to scavenge the ABTS radical. The differences in the ability to scavenge ABTS radical could be associated with the phenols’ content [8] and the active hydroxyl groups of the monosaccharides present in the polysaccharide [51,52].

The ability to scavenge the DPPH radical of GACP was lower compared with AA at concentrations between 0.1–0.8 mg/mL. The DPPH radical scavenging rate of AA was 97.16% and was close to that of GACP (97.09%) when the concentration reached 1.6 mg/mL (see Figure 4B). Comparing the IC_50_ value of *Ganoderma lucidum* (0.41 mg/mL) [27], and *Pleurotus ostreatus* (2.14 mg/mL) [53] polysaccharides, the IC_50_ value of GACP (0.36 ± 0.01 mg/mL) was lower, which indicated that GACP has better potential to scavenge the DPPH radical. It has been shown that the enhanced DPPH radical scavenging activity is related to the synergistic effect of polysaccharides and proteins [35,54], so it could be inferred that the presence of proteins in GACP contributed to its ability to scavenge this radical.

#### 2.3.2. Hydroxyl Radical (^•^OH) and Superoxide Radical (O_2_^−•^) Scavenging Ability

Figure 4C shown that the ^•^OH scavenging potential of AA and GACP increased in a concentration-dependent manner. Concentrations between 0.3 to 1.0 mg/mL GACP presented a better ^•^OH scavenging ability than AA and increased from 32.2 to 62.97%. IC_50_ value of GACP (0.67 ± 0.04 mg/mL) was higher than that reported for *Oudemansiella radicata* polysaccharides (0.5 mg/mL) [55], but lower compared to that reported for *Pleurotus ostreatus* (10.28 mg/mL) polysaccharides [53]. The number of active hydroxyl groups in a molecule can be related to the ^•^OH scavenging capacity [8,56]. Hence, the presence of phenols in GACP provides active hydroxyl groups that contribute to the high ^•^OH scavenging activity.

On the other hand, at a concentration range of 0.5–5 mg/mL, the O_2_^−•^ scavenging activity of GACP increased from 16.42 to 53.80%, while the O_2_^−•^ scavenging activity of AA increased from 51.85 to 98.02% (Figure 4D). Compared with the *Pleurotus ostreatus* (77.09 mg/mL) polysaccharide [57], the IC_50_ value of GACP (4.51 ± 0.18 mg/mL) was better, which demonstrates the ability of GACP to scavenge O_2_^−•^ radical. The O_2_^−•^ scavenging ability of polysaccharides could be associated with the electrophilic groups present in their structure, which can facilitate the hydrogen release from the O-H bond to stabilize the O_2_^−•^ radical. The energy required to dissociate the O-H bond will be lower the greater the number of electron-withdrawing groups attached to the polysaccharide. In this way, the O_2_^−•^ scavenging activity was enhanced by the presence of electrophilic groups such as aldehyde, sulfate, or keto [56,58,59]. The presence of sulfated sugars and uronic acids in GACP (see Table 1) provides the O_2_^−•^ scavenging activity, highlighting the potential use of GACP as a natural antioxidant.

#### 2.3.3. Reducing Power Assay

The reducing power is considered an indicator of the antioxidant activity of polysaccharides, due to the direct relationship between them [54]. Figure 4E shows the reducing power of AA and GACP. The reducing power of the GACP followed a concentration-dependent manner. At a concentration of 1.2 mg/mL, the AA presented an absorbance value of 2.61 while GACP was 0.53. At concentrations greater than 0.4 mg/mL, the absorbance value for AA oscillates from 2.40 to 2.60. In addition, the IC_50_ value of GACP (1.11 ± 0.04 mg/mL) was lower than that reported for *Ganoderma lucidum* (2.54 mg/mL) [27] and higher than *Pleurotus ostreatus* (0.28 mg/mL) polysaccharides [53]. According to the results, it could be suggested that the reducing power of GACP is related to the electron-donating ability of uronic acids and phenols linked to polysaccharides.

#### 2.3.4. Inhibition of Lipid Peroxidation

The antioxidant activity was also determined by the production of CDH and MDA. Table 4 shows the results of the inhibition of lipid peroxidation in MeLo. The results showed an antioxidant protective effect of GACP and BHT (positive control) compared with MeLo without preservative (negative control), in the initial and final stages of lipid peroxidation, which was evidenced in the low formation of CDH and MDA, respectively. The formation of CDH and MDA in the presence of GACP was similar to that observed for BHT. These results confirm the great potential of GACP to scavenge radicals derived from lipid peroxidation. The lipid radical scavenging could be associated with the sulfate and uronic acids, phenol, and protein content present in GACP. Our results are in agreement with those reported for polysaccharides from *Oudemansiella radicata* [60] and *Lentinula edodes* [61].

It is important to remark that different strains, cultivation techniques, development stages, storage conditions, and extraction methods, among others, cause the diversity and complexity of *Ganoderma* polysaccharides [30]. Likewise, the biological activity of polysaccharides is related to the chemical composition, molecular configuration, water solubility, branching degree, and molecular weight, among others [54,62]. Although *Ganoderma lucidum* is a well-known mushroom for its medicinal and health benefits, these properties will depend on the chemical composition [30]. Table 5 shows the compositional differences between *Ganoderma lucidum* polysaccharides (GLP) and GACP. Thus, the differences in the improved antioxidant activity of GLP and GACP could be associated with monosaccharide composition, molecular configuration, and the presence of uronic acids, sulfated sugars, proteins, and phenols, so it would not be necessary to carry out purification processes. This would represent lower costs for obtaining these extracts for their potential use in the food and pharmaceutical industry as a natural antioxidant.

### 2.4. Effect of GACP on Cell Viability and Cell Proliferation

**Figure 5:**
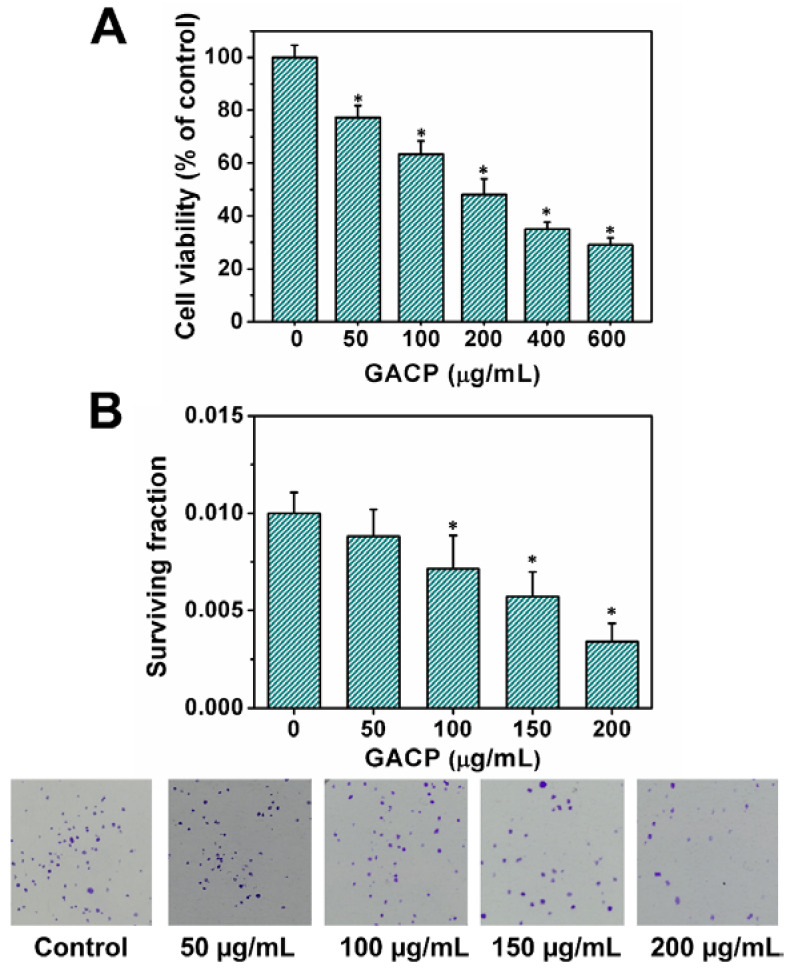
The effect of GACP on cell viability and cell proliferation: Cytotoxic effect on MG-G3 by MTT assay (**A**) and clonogenic assay (**B**) after 24 h of incubation. The values are presented as the mean ± SD (*n* = 3). *: statistically significant differences between control and treatment (*p* < 0.001).

Previous studies have shown that mushrooms’ polysaccharides have cytotoxic effects on different types of osteosarcoma cell lines [15,16,63]. Thus, the cytotoxic effect of GACP was evaluated on human MG-63 osteosarcoma cells by using the MTT assay and the results are shown in Figure 5A. The cell viability was decreased in a concentration-dependent manner upon exposure to increasing concentrations of GACP. IC_50_ value for GACP was 199.43 ± 9.46 μg/mL at 24 h and 126.64 ± 17.64 μg/mL at 72 h indicating that the cell viability also decreased in a time-dependent manner. Studies reporting cytotoxic effects of polysaccharides in human MG-63 osteosarcoma cells are incipient, however, it is notable that the obtained IC_50_ value of GACP at 24 h is within the range previously indicated for polysaccharides obtained from *Trametes robiniophila* Murrill, in U-2 OS human osteosarcoma cells (IC_50_ = 151.32 μg/mL) [16]. Likewise, a clonogenic assay was also performed to estimate the effect of GACP on cell reproductive potential. Figure 5B shows a reduction in cell proliferation in a concentration-dependent manner (0–200 μg/mL), which was in agreement with the cell viability results.

In the same way as antioxidant activity, the anticancer activity of polysaccharides may be associated with their molecular weight, chemical composition, and conformation. It has been shown that polysaccharides with a triple helical structure and antioxidant activity have high anticancer activity [44,64], which accorded with the structural features of GACP. Likewise, the immunomodulatory effect and the presence of (1→3)-β-glucans are factors that could influence the anticancer activity of polysaccharides [4,20,44,54].

ROS have an important influence on the generation and formation of cancer cells [65]. Since cancer cells grow with ROS levels that are moderately higher than those of normal cells, they may be sensitive to ROS level changes [66]. There are reports that polysaccharides can affect cancer cell growth by increasing the level of auto-oxidation and scavenging ROS [64,67]. In addition, although genuine apoptosis only occurs in the organism and is unviable in non-renewable cell classes commonly used in vitro, stress-induced apoptosis-like cell death (SIACD) is often confused with apoptosis in most in vitro studies carried out with cell lines [68,69,70]. Therefore, in the present study, we suggest that MG-63 cells experienced SIACD and not actual apoptosis. One way to decrease this stress may be that autophagy is activated as a secondary response either through ROS-mediated induction, or by increasing mitochondrial damage after treatment with polysaccharides [71,72,73,74]. Thus, the anticancer activity of GACP could be attributed to its ability to scavenge free radicals (^•^OH and O_2_^−•^). Although there are several mechanisms by which the anticancer activity of polysaccharides occurs [65,75], to fully understand the anticancer mechanism of GACP, further studies are needed.

## 3. Materials and Methods

### 3.1. Materials

*Ganoderma* aff. *australe* was collected in October 2019 in San Mateo, Boyacá, Colombia, at 2698 m above sea level (GPS coordinates 5°36’17.6″ N, 73°56’0.85″ W). A voucher specimen was stored at the herbarium of the University of Antioquia, Colombia (herbarium code: HUA-228455). This work was developed according to the Framework Contract for Access to Genetic Resources and their Derivative Products, 126 of 2016, RGE0156-13, Ministry of Environment and Sustainable Development, Colombia.

Acetone, acetonitrile, butanol, chloroform, ethanol, ether, Folin–Ciocalteu reagent, ferrous sulfate (FeSO_4_), hydrochloric acid (HCl), methanol, phenol, salicylic acid, sodium hydroxide (NaOH), and sulfuric acid, (H_2_SO_4_) were acquired from Merck Millipore (Darmstadt, Germany). Hydrogen peroxide (H_2_O_2_) and ascorbic acid (AA) were obtained from PanReac AppliChem (Darmstadt, Germany). The 2,2-azino-bis-(3-ethyl-benzthia-6-sulfonic acid) (ABTS), butylated hydroxytoluene (BHT), bovine serum albumin (BSA), chondroitin sulfate, carbazole, Congo red, 1,1-diphenyl-2-picrylhydrazyl (DPPH), 1,9-dimethyl-methylene blue (DMB), deuterium oxide (D_2_O), fucose (Fuc), galactose (Gal), gallic acid, glucose (Glc), glucuronic acid (GlcA), iron(III) chloride (FeCl_3_), mannose (Man), methyl linoleate (MeLo), 1-phenyl-3-methyl-5-pyrazolone (PMP), pyrogallol, potassium persulfate (K_2_S_2_O_8_), potassium ferricyanide (III) (K_3_[Fe(CN)_6_]), trifluoroacetic acid (TFA), 2-thiobarbituric acid (TBA), and tris(hydroxymethyl)aminomethane were purchased from Sigma-Aldrich (St. Louis, MO, USA.). Dulbecco’s modified Eagle’s medium (DMEM) and TrypLE^TM^ were obtained from Gibco (Gaithersburg, MD, USA). Fetal bovine serum (FBS) was acquired from Internegocios (Buenos Aires, Argentina). The 3-(4,5-dimethylthiazol-2-yl)-2,5-diphenyltetrazolium bromide (MTT) was obtained from Invitrogen Corporation (Buenos Aires, Argentina). All reagents used were of analytical grade.

### 3.2. Extraction of Polysaccharides

The extraction of water-soluble polysaccharides was performed according to Sun et al. (2014) and Zhao et al. (2015) with some modifications [15,16]. The dried fruiting bodies were subjected to solvent extraction for 3 h, using ethanol to remove polyphenols, pigments, lipids, and other small molecules. The residue obtained was again subjected to solvent extraction with distilled water at 95 °C for 3 h each time, threefold (ratio 1:20 w/v = residue: distilled water). Then, the supernatant was concentrated under reduced pressure in a rotary evaporator at 50 °C and deproteinized using Sevag reagent (1:4, v/v = n-butanol: chloroform) for 30 min (5:1, v/v Sevag reagent: polysaccharide solution). Subsequently, the resulting solution was centrifuged at 3500 rpm for 15 min, twice. The aqueous supernatant was left overnight in ethanol at 4 °C (5:1, v/v = ethanol: polysaccharide extract), and the precipitated polysaccharides were obtained by centrifugation at 3500 rpm for 15 min. Ethanol, acetone, and ether were used to wash the polysaccharide-enriched extract, and the process was carried out twice. The final polysaccharides were dissolved in distilled water and taken to dialysis on a membrane (MWCO12-14 KDa) for 48 h. The final solution was lyophilized to obtain *G.* aff. *australe* crude polysaccharides (GACP). In addition, the ultracentrifugation process was utilized to estimate the molecular weight of GACPs (100, 50, and 10 KDa MWCO filters).

### 3.3. Characterization of GACP

#### 3.3.1. Chemical Composition

The neutral content of sugar was determined at 490 nm, by the phenol–sulfuric acid method, using glucose as standard (y = 0.010x − 0.006, R^2^ = 0.999) [76]. A total of 30 µL of GACP (1 mg/mL) and 370 µL of distilled water were mixed. Then, 2000 µL of H_2_SO_4_ was added, and the mixture was stirred. After, 400 µL of 5% w/v phenol was added and stirred. The mixture was placed at 90 °C for 5 min, cooled and the absorbance value was measured. The uronic acid content was analyzed at 525 nm by the carbazole–sulfuric acid method with glucuronic acid as a standard (y = 0.008x + 0.027, R^2^ = 0.996) [77]. A total of 80 µL of GACP (1 mg/mL) and 320 µL of distilled water were mixed, and 2000 µL of 0.095% w/v sodium tetraborate in H_2_SO_4_ was added. The mixture was shaken, placed at 100 °C for 12 min, and 40 µL of 0.2% w/v carbazole ethanolic solution was added. Next, the mixture was shaken, placed at 100 °C for 10 min and cooled. The sulfated sugar content was measured at 520 nm using the reported dimethyl methylene blue method with chondroitin sulfate as a standard (y = 0.006x + 0.011, R^2^ = 0.997) [78]. Then, 100 µL of GACP (1 mg/mL) and 400 µL of distilled water were mixed. After that, 3000 µL of DMB in sodium acetate (0.05 M, pH 4.7) (0.0011% w/v) was added and the mixture was stirred during 15 s. The mixture was placed in the darkness for 30 min and the absorbance value was measured.

The Bradford method was used to analyze the protein content at 595 nm with bovine serum albumin as a standard (y = 0.002x + 0.001, R^2^ = 0.998)) [79]. A total of 750 µL of Bradford reagent and 250 µL of GACP (1 mg/mL) were mixed and placed for 5 min in the darkness. Finally, the absorbance value was measured. The total phenols’ content was analyzed at 765 nm using the reported Folin–Ciocalteu method with gallic acid as standard (y = 0.088x − 0.012, R^2^ = 0.996) [22]. Briefly, 1500 µL of distilled water, 100 µL of 1 mg/mL GACP, and 100 µL of Folin–Ciocalteu reagent were mixed and stood at room temperature for 10 min. After that, 300 µL of 20% w/v sodium carbonate was added to the mixture and incubated at 40 °C for 30 min. The mixtures were cooled, and the absorbance value was measured.

#### 3.3.2. Monosaccharide Analysis

High-performance liquid chromatography with diode array detector (HPLC-DAD) and PMP derivatization were used to determine the monosaccharide composition of GACP [22]. A total of 5 mg of GACP was dissolved in 2000 µL of 2 M TFA and incubated at 110 °C for 4 h. The hydrolysate was dried under reduced pressure at 50 °C. The residue was dissolved in methanol and then it was evaporated to dryness. This step was repeated four times to remove the TFA residual [80] and the residue was dissolved in 2000 µL of distilled water. Then, 450 µL of 0.5 M PMP methanolic solution was mixed with 450 µL of hydrolysate solution and 450 µL of 0.3 M NaOH and incubated at 70 °C for 30 min. After that, the reaction was stopped by the addition of 450 µL of 0.3 M HCl, and the mixture was extracted with chloroform three times. The aqueous product was filtered through a 0.45 µM membrane and analyzed by HPLC (LC 300 HPLC System) equipped with DAD detectors and a C18 column (Pinnacle, 250 mm × 4.6 mm). The mobile phase was composed of potassium phosphate buffer saline solution (0.05 M, pH 6.9) and acetonitrile in a ratio of 83:17 (v/v), respectively, and the UV detector wavelength was 250 nm. The standard sugars were derivatized likewise.

#### 3.3.3. Congo Red Assay

The Congo red assay was determined according to Dong et al., with some modifications [45]. A total of 500 µL of 1 mg/mL GACP (1 mg/mL) and 500 µL of 80 mM Congo red solution were mixed, and then different volumes of distilled water and a solution of 1 mol/L NaOH were added to adjust the final concentration of NaOH (0–0.5 mol/L). The mixture of Congo red and distilled water without polysaccharides was used as a control. After 5 min, the maximum wavelength absorption (λ_max_) between 400 and 600 nm was recorded.

#### 3.3.4. UV–Vis and Fourier-Transform Infrared Spectroscopy Analysis (FT-IR)

The UV–Vis spectrum of the GACP aqueous solution (0.005 mg/mL) was recorded in a Thermo Scientific Evolution 60S UV–Visible spectrophotometer (Thermo Scientific, Waltham, MA, USA), in the range of 200–500 nm. The FT-IR spectrum was determined by attenuated total reflectance (ATR) technique in a PerkinElmer Spectrum Two Spectrometer (PerkinElmer, Waltham, MA, USA) at the frequency range from 500 to 4000 cm^−1^.

#### 3.3.5. Nuclear Magnetic Resonance Analysis (NMR)

A Bruker Ascend III HD 600 MHz spectrometer (Bruker, Billerica, MA, USA) operating at 600 MHz for ^1^H and 150 MHz for ^13^C was used to obtain ^1^H, DEPT-135, and ^1^H-^13^C-HSQC spectra. All spectra were taken with HOD pre-saturation suppression at 30 °C, using deuterated acetone as internal standard at 2.04 ppm for ^1^H. Then, 30 mg of GACP was dissolved in 500 µL D_2_O.

### 3.4. Antioxidant Activity

#### 3.4.1. ABTS and DPPH Radical Scavenging Assay

The ABTS radical scavenging potential was evaluated with the reported method by Gu et al. with minor modifications [9]. A total of 2000 µL of K_2_S_2_O_8_ (2.45 mM) was mixed with 2000 µL of ABTS (7 mM) and incubated at room temperature in the dark for 12–16 h. Distilled water was used to dilute the ABTS solution to an absorbance of 0.70 ± 0.02 at 734 nm. A total of 1000 µL of ABTS diluted solution was mixed with 100 µL of different GACP concentrations (0–3 mg/mL), and then incubated for 6 min in the darkness. The absorbance value at 734 nm was measured. The DPPH radical scavenging potential was assessed according to the reported method by Zhu et al., with slight modifications [40]. Then, 1000 µL of 0.05 mM DPPH methanolic solution was mixed with 200 µL of different GACP concentrations (0–4 mg/mL). The mixture was shaken and placed for 30 min in the dark. The absorbance value at 517 nm was determined.

#### 3.4.2. Hydroxyl Radical (^•^OH) and Superoxide Radical (O_2_^−•^) Scavenging Assay

The ^•^OH scavenging potential was measured according to Gu et al., with some modifications [9]. A total of 100 µL of different GACP concentrations (0–3 mg/mL), 500 µL of 0.15 mM FeSO_4_, 500 µL of 6 mM H_2_O_2_, 200 µL of 2 mM salicylic acid in ethanol, and 200 µL distilled water were mixed. The mixtures were incubated at 37 °C for 1 h, and the absorbance value at 510 nm was measured. On the other hand, the O_2_^−•^ scavenging potential was evaluated by the reported method by Shang et al. with minor modifications [81]. A mixture composed of 1125 µL of 50 mM Tris-HCl buffer at pH 8.2 and 125 µL at different GACP concentrations (0–10 mg/mL) was incubated for 20 min at 25 °C. Then, 250 µL of 25 mM pyrogallol was added to the mixture and incubated for 4 min at 25 °C. To stop the reaction, 250 µL of 8 mM HCl was added. The absorbance value at 325 nm was determined.

#### 3.4.3. Reducing Power Assay

The reducing power was determined according to the method of Hamed et al., with some modifications [51]. A total of 625 µL of 200 mM phosphate buffer at pH 6.6, 250 µL of different GACP concentrations (0–4 mg/mL), and 625 µL of 1% (w/v) K_3_[Fe(CN)_6_] were mixed and incubated at 50 °C for 30 min. Later, 625 µL was added and the mixture was centrifuged 10 min at 3000 rpm. Then, 625 µL of supernatant, 625 µL of distilled water and 125 µL FeCl_3_ (1% w/v) were mixed and placed in the darkness for 10 min. The absorbance value at 700 nm was determined. AA was used as a control. The IC_50_ value refers to the compound concentration in which 50% inhibitory activity is obtained [82]. The graph of radical scavenging ability percentage against samples’ concentrations was used to obtain IC_50_ values.

#### 3.4.4. Determination of Conjugated Diene Hydroperoxide (CDH) and Thiobarbituric Acid Reactive Substances (TBARS)

In addition, the potential to inhibit the lipid peroxidation in MeLo was carried out according to Mejía-Giraldo et al. [83]. The CDH concentration produced was expressed as mmol CDH/kg MeLo. A total of 100 µL of 2 mg/mL GACP or BHT and 900 µL of 10 mM MeLo were mixed and placed at 40 °C for 5 days. Next, 1000 µL of ethanol was used to dissolve each sample. The dissolved sample was diluted in ethanol (1:25 v/v) and the absorbance value was measured at 234 nm. The extinction coefficient was 29,000/M × m.

The TBARS content was also determined and expressed as mmol malondialdehyde (MDA)/kg MeLo. A total of 50 µL of the dissolved sample, 350 µL of ethanol, 100 µL of BHT-ethanol, and 500 µL of 0.37% TBA in 0.25 mM HCl were mixed and incubated for 30 min at 90 °C. The mixtures were cooled and centrifugated for 10 min at 3000 rpm. The absorbance values at 535 nm were determined and corrected for non-specific turbidity by subtracting the absorbance at 600 nm. We used 156,000/M × cm used as the molar extinction coefficient. MeLo and BHT were used as a control.

### 3.5. Culture of Human MG-63 Osteosarcoma Cells

Human MG-63 osteosarcoma cells were obtained from American Type Culture Collection (ATCC^®^, CRL-1427; Manassas, VA, USA). MG-63 cells were grown in DMEM containing 10% fetal bovine serum (FBS), 100 IU/mL penicillin, and 100 µL/mL streptomycin in a 5% CO_2_ atmosphere at 37 °C. A 75 cm^2^ flask was used to seed the MG-63 cell line until reaching 70–80% of confluence. Cells were sub-cultured using TrypLE and were grown in multi-well plates. After 24 h, the monolayers were incubated under different conditions according to the experiments.

### 3.6. Cell Viability by MTT Assay

The effect of GACP on the viability of MG-63 cells was evaluated by the reported method described by Mosmann [84]. Cells were seeded in a 96-well dish and allowed to attach for 24 h. After, cells were treated with different GACP concentrations (0, 50, 100, 200, 400, and 600 µg/mL) at 37 °C for 24 h. Then, the supernatant was removed, 100 μL of 0.5 mg/mL MTT solution was added, and cells were cultured for 3 h, 100 μL of DMSO was added to each well, and the absorbance value at 570 nm was measured in a multiplate reader Multiskan FC, (Thermo Fisher Scientific, Waltham, MA, USA). The GACP concentration providing 50% of inhibitory activity (IC_50_) was obtained from the graph of cell viability percentage against GACP concentration.

### 3.7. Clonogenic Assay

The effect of GACP on cell proliferation was determined according to the method described by Balsa et al., with some modifications [85]. MG-63 human osteosarcoma cells were grown in a 24-well plate. Cells were exposed to different GACP concentrations (0, 50, 100, 150, and 200 µg/mL) for 24 h. After, the cells were washed with PBS, and 1500 μL of DMEM-10% FBS were added. The plates were incubated for 8 days. Then, the cells were washed with PBS and stained with a mixture of 0.5% of crystal violet and 6% of glutaraldehyde and at room temperature for 30 min. Finally, the plates were washed and dried to count the colonies. The plating efficiency (PE) and the surviving fraction (SF) were calculated as:

(1) $$\mathrm{PE}\;\left(\%\right)=\frac{\mathrm{the}\;\mathrm{number}\;\mathrm{of}\;\mathrm{colonies}\;}{\mathrm{the}\;\mathrm{number}\;\mathrm{of}\;\mathrm{cells}\;\mathrm{seeded}\;}\times 100$$

(2) $$\mathrm{SF}\;\left(\%\right)=\frac{\;\mathrm{the}\;\mathrm{number}\;\mathrm{of}\;\mathrm{colonies}\;\mathrm{that}\;\mathrm{survive}\;\mathrm{after}\;\mathrm{treatment}}{\mathrm{PE}\times \;\mathrm{the}\;\mathrm{number}\;\mathrm{of}\;\mathrm{cells}\;\mathrm{seeded}}$$

### 3.8. Statistical Analysis

Each assay was carried out by triplicate, and results were reported as mean ± standard deviation (SD). Statistical differences were analyzed using the analysis of variance method (ANOVA) (*p* < 0.05).

## 4. Conclusions

In this work, we investigated and reported for the first time the biological activity of the crude extract of polysaccharides isolated from *G.* aff. *australe* collected in the Colombian Andes. The results of chemical characterization showed that GACP contained α and β-D-glucopyranosyl residues, and also exhibited triple helical conformation at low NaOH concentrations. The higher antioxidant capacity of GACP was related to the synergistic effect of the presence of compounds such as uronic acids, sulfated sugars, phenols, and proteins in the polysaccharide extract. Furthermore, GACP showed cytotoxic effects and inhibited cell proliferation of human MG-63 osteosarcoma cells. The biological activity of GACP is related to its structural characteristics and its chemical composition. This work demonstrated the importance of the use and cultivation of wild mushrooms for the development of compounds with biological activity and their potential application as an alternative for osteosarcoma treatment.

## Figures and Tables

**Table 1:** Results of chemical composition for GACP.

Sample	GACP
Neutral sugar (%)	79.31 ± 3.05
Uronic acid (%)	8.98 ± 1.61
Sulfated sugar (%)	6.42 ± 0.38
Protein (%)	2.91 ± 0.18
Total polyphenols (%)	3.49 ± 0.06
Monosaccharide components	Man, GlcA, Glc, Gal and Fuc

Man (mannose); GlcA (glucuronic acid); Glc (glucose); Gal (galactose); and Fuc (fucose).

**Table 2:** ^13^C and ^1^H NMR chemical shifts of GACP.

Sugar Residues		Chemical Shifts (ppm)					
		H1/C1	H2/C2	H3/C3	H4/C4	H5/C5	H6a, H6b/C6
A	β-D-Glcp-(1→	4.46	3.26	3.42	3.39	3.56	3.83, 3.67
		102.41	73.17	75.12	69.66	74.34	60.69
B	→3)-β-D-Glcp-(1→	4.68	-	3.69	3.42	3.83	3.97
		102.41	-	84.08	69.27	79.41	70.05
C	α-D-Glcp(1→	4.93	-	3.66	3.33	3.91	-
		97.34	-	73.09	71.06	73.97	-

-: No determined.

**Table 3:** IC_50_ values of AA and GACP on antiradical activities.

Crude Extract	IC_50_ (mg/mL)				
	ABTS	DPPH	Hydroxyl Radical	Superoxide Radical	Reducing Power
AA	0.04 ± 0.001	0.01 ± 0.001	0.79 ± 0.02	0.46 ± 0.03	0.042 ± 0.002
GACP	0.48 ± 0.03	0.36 ± 0.01	0.67 ± 0.04	4.51 ± 0.18	1.11 ± 0.04

AA: ascorbic acid; ABTS: 2,2-azino-bis-(3-ethyl-benzthia-6-sulfonic acid); DPPH: 1,1-diphenyl-2-picrylhydrazyl.

**Table 4:** Inhibition of lipid oxidation of MeLo by GACP.

Samples	CDH	MDA
	mmol/Kg MeLo	
MeLo	163.72 ± 10.16	10.42 ± 0.95
BHT	30.68 ± 3.43 *	0.06 ± 0.01
GACP	7.00 ± 0.61 *	0.07 ± 0.02

*: statistically significant differences between BHT and GACP (*p* < 0.001). BHT: butylated hydroxytoluene; CDH: conjugated diene hydroperoxide; MDA: malondialdehyde; MeLo: methyl linoleate.

**Table 5:** Comparison of conformation and chemical composition of GACP and GLP.

Sample	GACP	GLP
Neutral sugar (%)	79.31	84.5
Uronic acid (%)	8.98	14.41
Sulfated sugar (%)	6.42	-
Protein (%)	2.91	-
Total polyphenols (%)	3.49	0.43
Monosaccharide components	Man, GlcA, Glc, Gal, and Fuc	Man, Rha, Glc, Gal, and Ara
α and β glycosidic bonds	Yes	Yes
Triple helical conformation	Yes	No
Reference	This work	[27]

Man: mannose; GlcA: glucuronic acid; Rha: rhamnose; Glc: glucose; Gal: galactose; Fuc: fucose; and Ara: arabinose.

## Data Availability

The data presented in this study are available in the article.
